# Altitudinal gradient affect abundance, diversity and metabolic footprint of soil nematodes in Banihal-Pass of Pir-Panjal mountain range

**DOI:** 10.1038/s41598-021-95651-x

**Published:** 2021-08-10

**Authors:** Shahid Afzal, Humira Nesar, Zarrin Imran, Wasim Ahmad

**Affiliations:** Nematode Biodiversity Research Lab, Department of Zoology, Aligarh Muslim University, Aligarh, 202002 India

**Keywords:** Ecology, Zoology

## Abstract

Despite enormous diversity, abundance and their role in ecosystem processes, little is known about how community structures of soil-inhabiting nematodes differ across elevation gradient. For this, soil nematode communities were investigated along an elevation gradient of 1000–2500 masl across a temperate vegetation in Banihal-Pass of Pir-Panjal mountain range. We aimed to determine how the elevation gradient affect the nematode community structure, diversity and contribution to belowground carbon assimilation in the form of metabolic footprint. Our results showed that total nematode abundance and the abundance of different trophic groups (fungivores, herbivores and omnivores) declined with the increase of elevation. Shannon index, generic richness and evenness index indicated that nematode communities were more diverse at lower elevations and declined significantly with increase in elevation. Nematode community showed a pattern of decline in overall metabolic footprint with the increase of elevation. Nematode abundance and diversity proved to be more sensitive to elevation induced changes as more abundant and diverse nematode assemblage are supported at lower elevations. Overall it appears nematode abundance, diversity and contribution to belowground carbon cycling is stronger at lower elevations and gradually keep declining towards higher elevations under temperate vegetation cover in Banihal-pass of Pir-Panjal mountain range.

## Introduction

Soils harbour some of the most diverse microbial community on earth, provide shelter to 25% world-wide described species and thus considered as a crucial biodiversity reservoir^1–6^. Soil organism’s distribution patterns play critical roles in determining the above and belowground primary production and its composition^7,8^. Elevation and its related factors significantly affect the soil abiotic characteristics and the density/biomass patterns of nematode communities, thus alter the microbial functions of soil ecosystem^9–12^.

All chemical and physical changes in the soil are rapidly reflected through changes in richness and composition of nematode species^13^. In addition to diversity and functional indices which are useful descriptive tools for assessment of food web and ecosystem condition, various metabolic footprint indices^14^ have been developed to estimate contribution of nematodes to various ecosystem services and functions. Among the landscape properties, altitude rather than vegetation cover is found to have vital effect on nematode community^15^, because altitudinal climatic conditions strongly constrain the availability and turnover of basal resources and can be viewed as nature’s own field experiments^16^. Various elevation patterns of diversity have been studied across a wide range of taxonomic groups in aboveground organisms, including trees, mammals, birds, insects and amphibians^17^. However, elevational diversity patterns of belowground organisms, especially those of soil invertebrates which often represent decomposer subsystems, have remained understudied^4,18^. Few studies have highlighted the effect of elevation on nematode biodiversity and community structure; suggesting a mid-elevation maximum diversity^19^, increase in diversity with elevation^20^, decrease in abundance with elevation^21,22^ and no change in abundance^23,24^. Therefore, there are no obvious trend in soil nematode diversity and abundance with regards to elevation. Thus, there is a need to further investigate mountain ecosystems in order to gain insights into the effect of elevation on diversity and abundance patterns of soil nematodes.

In the present study, we tried to unravel the distribution and contribution of soil dwelling nematodes to belowground soil food web from 1000 to 2500 masl elevation gradient along a pristine temperate forest vegetation cover at Banihal-Pass of Pir-Panjal mountain range which lies in the Western Himalayan region. The climate at higher elevations of the mountain range are harsher with varying temperatures and precipitation received in the form of snow and rain. Therefore, we studied nematodes which are considered as ideal bio-indicators for terrestrial ecosystems and their community assemblages can provide critical insights regarding many aspects of ecosystem functions. Further findings from this study can be used as basic information for additional research that will be applied to investigate the soil biota in this mountain range. We assessed total and trophic abundance, biomass, alpha diversity, and metabolic footprint pattern of soil nematodes along the elevation gradient. Since the abiotic factors shape species distributions, fostering fewer species and abundance at high elevations may be due to harsh climatic conditions compared to low elevations. Keeping this in consideration, we hypothesized: (1) there will be environmental filtering increasing towards higher elevations due to low temperature and frequent temperature fluctuations. In addition, the mountain ecosystems are generally subject to more disturbances, due to rain wash in sparse vegetated soils, frequent frost and frequent freezing and thawing. We therefore predicted that these factors would shape a declining pattern in nematode abundance and diversity with increasing elevation and (2) as metabolic rates are closely related with temperature, we expected that the metabolic footprint of nematodes would be suitable indicators of elevation change, we therefore hypothesized that metabolic footprint will decrease with increasing altitude.

## Results

### Physicochemical properties of soil along elevation gradient

**Figure 1:**
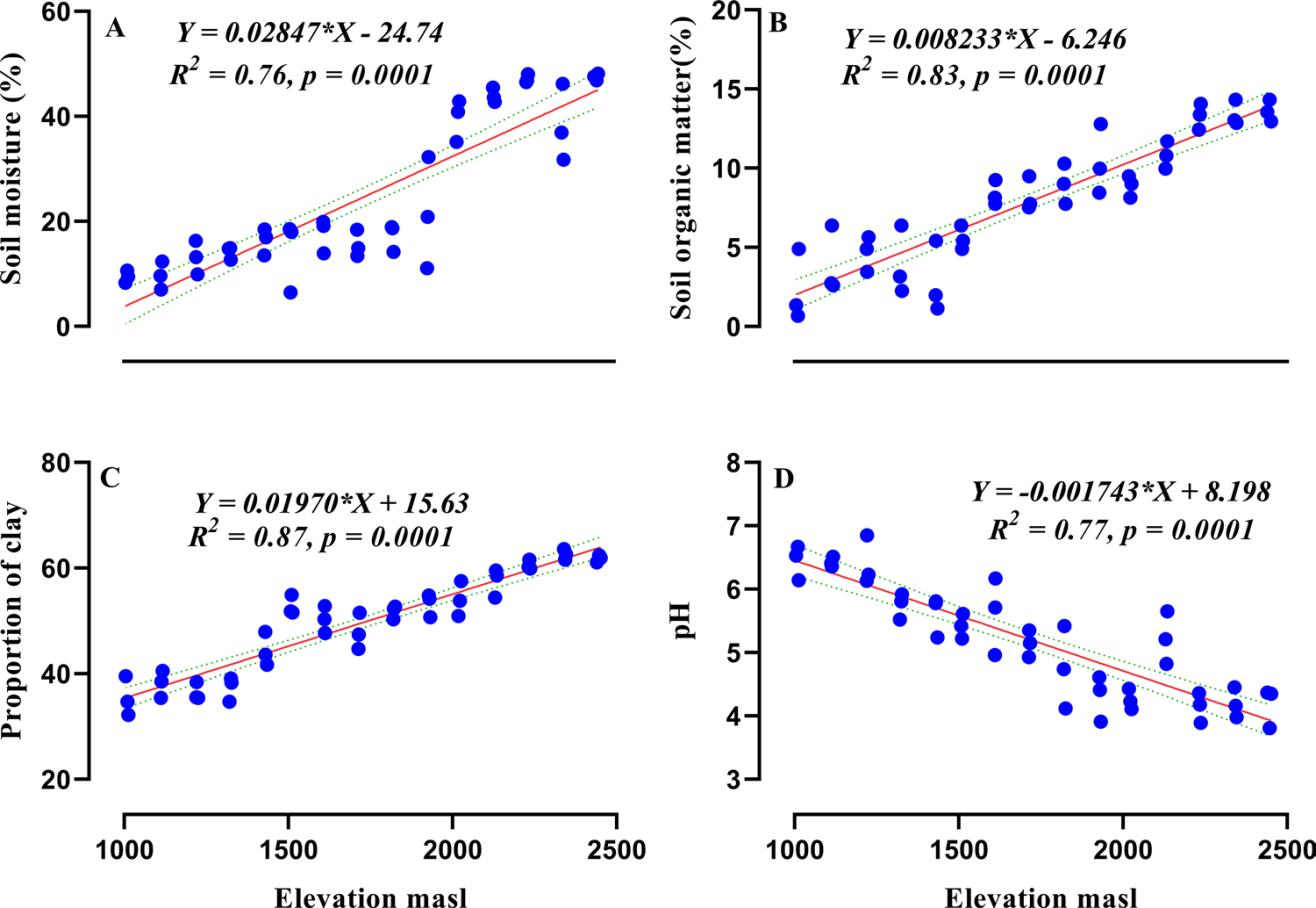
Effect of elevation on soil moisture (**A**), soil organic matter (**B**), proportion of clay (**C**) and pH (**D**). Equation for *Y*, *R*^2^ (coefficient of determination), *p* value of linear regression line and dotted lines of 95% of confidence interval are shown.

Among the abiotic factors (Table 1), soil moisture (r = 0.87, *p* = 0.0001; Fig. 1A), soil organic matter (r = 0.92, *p* = 0.0001; Fig. 1B) and proportion of clay (r = 0.92, *p* = 0.0001; Fig. 1C) showed significant positive correlation with elevation. Whereas, soil pH (r = − 0.87, *p* = 0.0001; Fig. 1D) significantly declined with increasing elevation.

**Table 1:** Slope coefficients, *p* values and standard errors (SE) for linear regressions showing the relationships between elevation and abiotic factors, nematode abundance, biomass, ecological indices and metabolic footprint.

Category	Variables	Slope	*p* value	SE
Abiotic factors	Soil moisture	0.02847	< 0.0001	0.0023
	Soil organic matter	0.008233	< 0.0001	0.0005
	Clay proportion	0.01970	< 0.0001	0.0011
	Soil pH	− 0.00174	< 0.0001	0.0001
Abundance	Total nematodes	− 0.5068	0.0042	0.1676
	Bacterivores	− 0.1713	0.0530	0.0821
	Fungivores	− 0.1707	0.0231	0.0724
	Herbivores	− 0.1067	0.0099	0.0395
	Omnivores	− 0.02952	0.0052	0.0100
	Predators	− 0.02851	0.1003	0.0169
Biomass	Total nematodes	− 0.00042	0.0042	0.0001
	Bacterivores	− 8.93100	0.0530	4.8390
	Fungivores	− 5.78500	0.0231	2.4500
	Omnivores	− 0.06194	0.0099	0.0209
	Predators	− 0.00011	0.0052	8.3780
Ecological indices	Shannon index (*H*)	− 0.00046	0.0004	0.0001
	Generic richness (Chao1)	− 0.00262	0.0216	0.0010
	Evenness (*J*)	− 0.00012	0.0024	3.8860
	Sum of Maturity index (SMI)	− 0.00016	0.1046	9.7500
	Maturity index (MI)	− 0.00028	0.0033	9.1400
	Plant parasitic index (PPI)	− 0.00038	0.0025	0.0001
	Basal index (BI)	0.0053	0.2941	0.0049
	Enrichment index (EI)	− 0.00597	0.3811	0.0067
	Structured index (SI)	− 0.02147	0.0014	0.0062
Metabolic footprint	Composite	− 0.1347	0.0013	0.0390
	Enrichment	− 0.02629	0.0196	0.0108
	Structure	− 0.04426	0.0260	0.0192

### Changes in abundance and biomass of nematode community along elevation gradient

**Figure 2:**
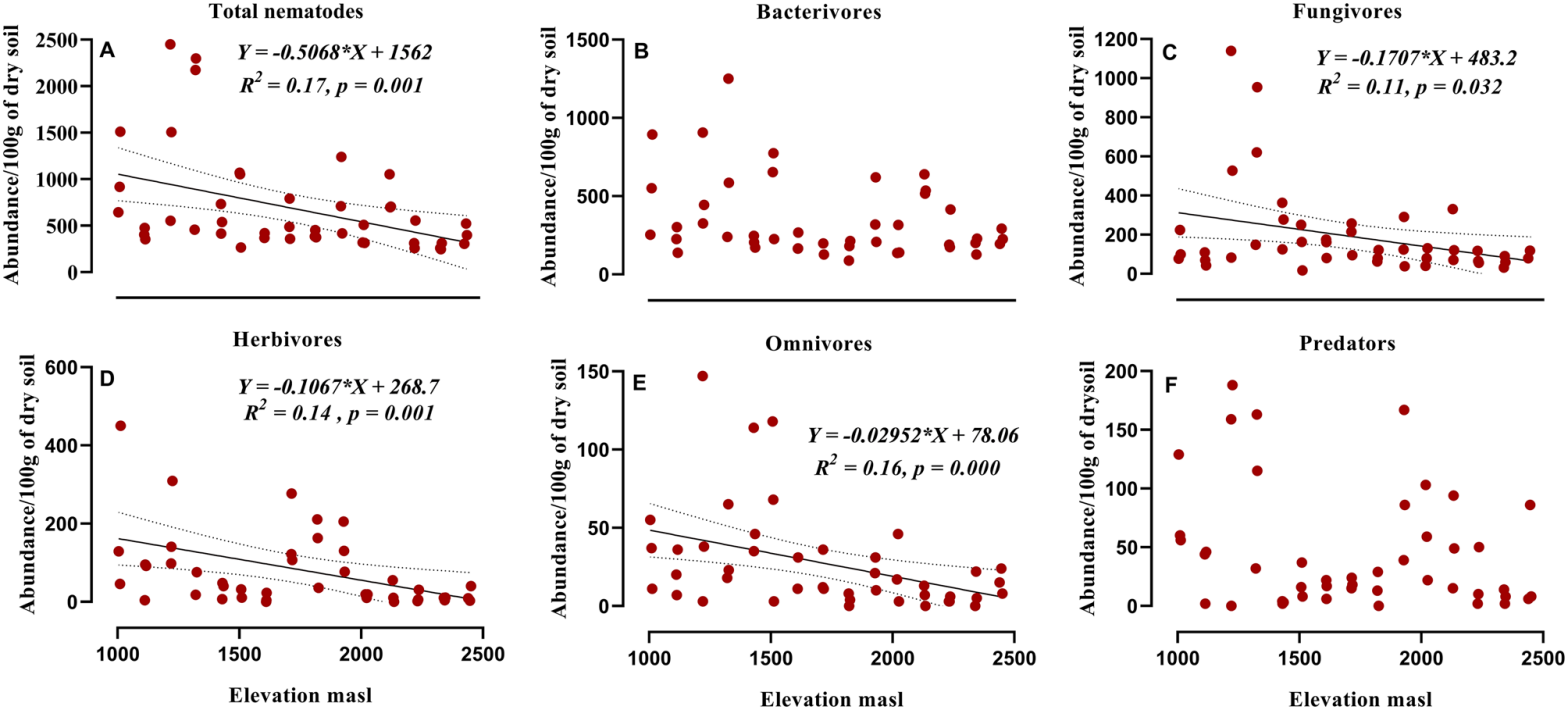
Relationship between elevation and total abundance of nematodes (**A**), trophic groups: Bacterivores (**B**), Fungivores (**C**), Herbivores (**D**), Omnivore (**E**), Predators (**F**). Equation for *Y*, *R*^2^ (coefficient of determination), *p* (significance) of linear regression and regression line and dotted lines of 95% of confidence interval are shown only for significant relationships.

**Figure 3:**
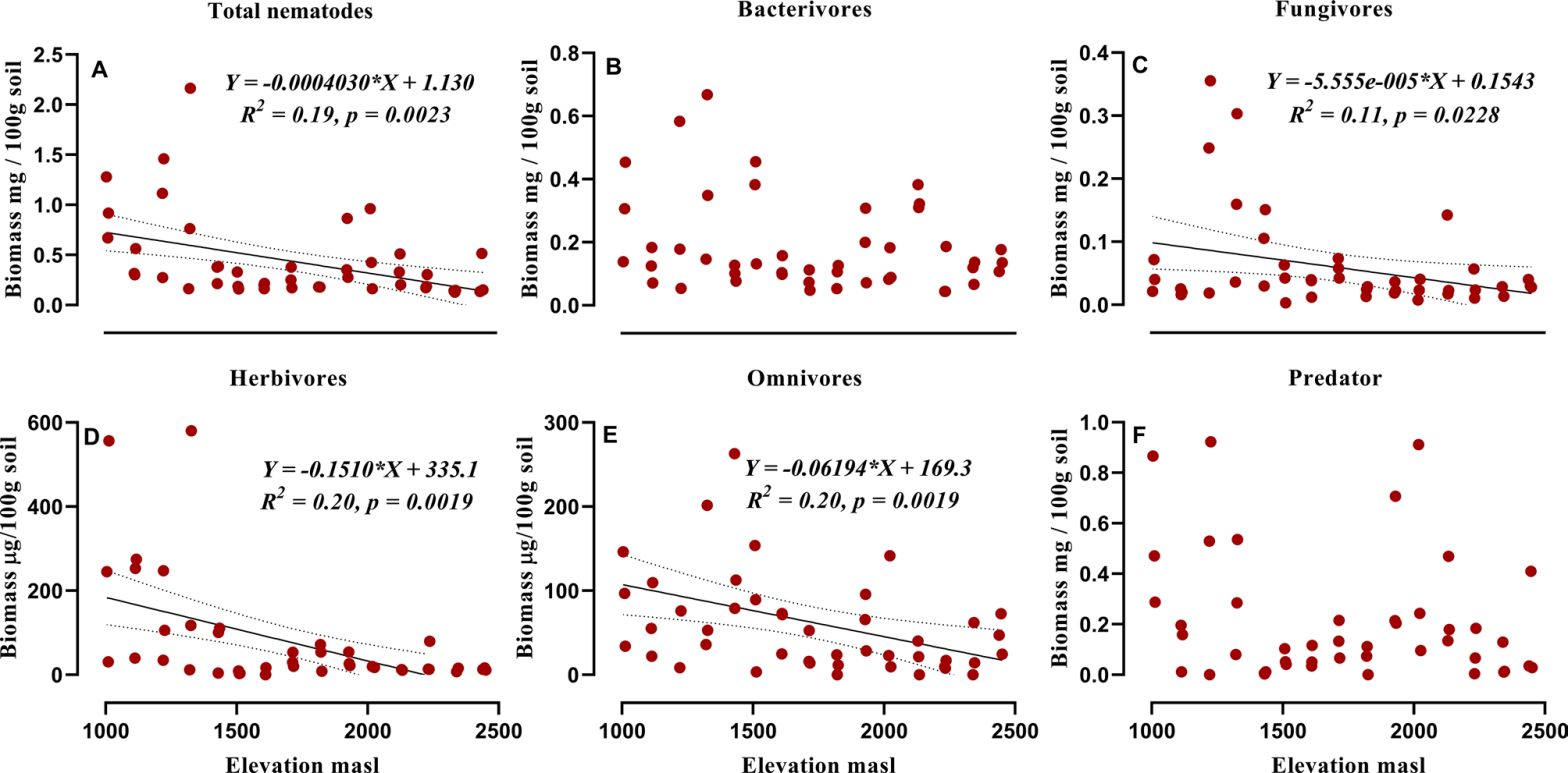
Relationship between elevation and total biomass of nematodes (**A**), biomass of trophic groups: Bacterivores (**B**), Fungivores (**C**), Herbivores (**D**), Omnivores (**E**), Predators (**F**). Equation for *Y*, *R*^2^ (coefficient of determination), *p* (significance) of linear regression, regression line and dotted lines of 95% of confidence interval are displayed only for significant relationships.

**Figure 4﻿:**
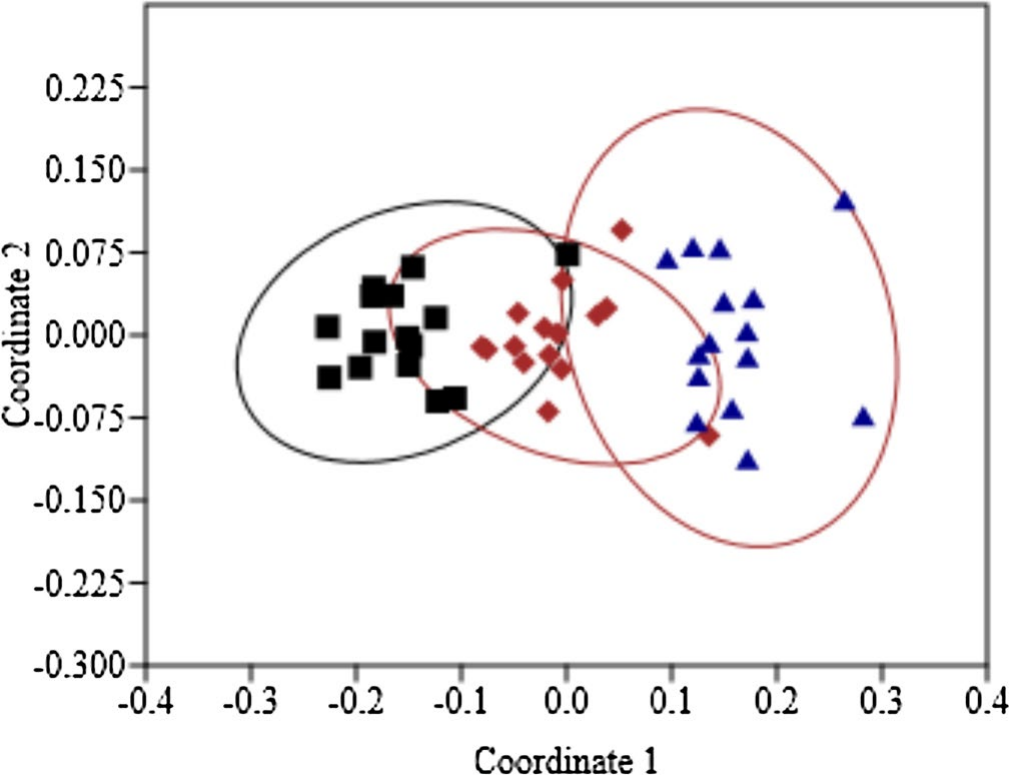
Non-metric multidimensional scaling (NMDS) ordination plot based on Bray–Curtis similarity measure of the nematode community between elevations. Squares represent 1000–1500 m; diamond represent 1500–2000 m and triangle represent 2000–2500 m. The circles surrounding the clusters represent 95% confidence intervals.

The total nematode abundance, biomass and diversity were correlated and tested with simple linear regression with elevation (Table 1). From 45 soil samples, 30,955 nematodes recovered were assigned to 49 different genera (Table 2) in five different trophic groups (bacterivore-12, fungivore-12, omnivore-4, predatory-8 and herbivore-13). Total nematode abundance (Fig. 2A) as well as abundance of fungivores, omnivores and herbivores (Fig. 2C–E) were negatively correlated with the increase of elevation, whereas, the abundance of bacterivores and predators (Fig. 2B,F) did not show any definite pattern. Similarly, the total nematode biomass (Fig. 3A) and biomass of the three trophic groups decreased with elevation (Fig. 3C–E). However, the biomass of both bacterivore and predatory nematodes (Fig. 3B,F) did not show any significant decline with elevation. Nematode communities significantly changed with elevation. The NMDS (Fig. 4) ordination showed three distinct clusters mainly separated by elevation level, which revealed that different elevation zones provide shelter to varying abundances of nematode communities (ANOSIM test, Global *R* = 0.74,* P* = 0.001). Overall, separation between elevations showed good strength, with the stress value of 0.10.

**Table 2:** Mean relative abundance of nematode genera (%) along elevation gradient. (n = 3).

Genus	Functional guild^b^	Elevation^a^														
		1000 m	1100 m	1200 m	1300 m	1400 m	1500 m	1600 m	1700 m	1800 m	1900 m	2000 m	2100 m	2200 m	2300 m	2400 m
**Bacterivores**																
*Acrobeles*	Ba2	0.0	0.0	2.5	0.0	5.3	0.0	0.0	6.4	0.0	20.4	0.0	0.0	26.1	1.3	0.2
*Acrobeloides*	Ba2	21.0	40.4	20.8	32.5	10.7	52.0	43.7	5.4	32.5	10.6	46.0	59.6	20.5	48.3	34.2
*Alaimus*	Ba4	3.9	1.6	0.5	2.8	2.1	0.1	1.1	0.1	0.0	0.2	1.3	0.0	17.6	0.2	0.0
*Cephalobus*	Ba2	3.9	2.7	2.0	2.8	5.1	0.7	1.0	2.1	2.4	1.9	0.4	3.7	2.5	1.2	1.2
*Chiloplacus*	Ba2	0.0	0.0	0.0	0.0	0.0	0.4	1.5	0.0	0.0	0.0	0.0	0.0	0.0	0.0	0.0
*Diplogaster*	Ba1	0.0	0.0	8.7	0.0	0.0	0.0	0.0	0.0	0.0	0.4	0.0	0.0	0.0	0.0	0.0
*Eucephalobus*	Ba2	0.4	0.0	0.0	0.0	0.1	0.8	0.0	0.0	0.0	0.0	0.0	0.0	0.0	2.6	4.3
*Mesorhabditis*	Ba1	12.4	2.8	1.5	1.1	10.7	14.6	1.2	9.6	3.1	2.6	2.0	1.1	1.2	11.9	12.6
*Monhystera*	Ba2	0.1	0.0	0.0	0.0	0.0	0.4	0.0	0.0	0.0	0.0	0.0	0.0	0.0	0.0	0.0
*Plectus*	Ba2	1.7	2.7	3.9	1.8	2.8	0.4	3.0	3.9	1.9	12.7	2.0	4.5	0.7	2.9	5.7
*Prismatolaimus*	Ba3	1.6	3.2	0.2	1.1	0.0	0.0	0.0	0.0	0.0	0.0	0.2	0.0	0.4	0.0	0.0
*Wilsonema*	Ba2	0.2	0.6	0.2	0.0	0.2	0.0	0.0	0.0	0.0	0.0	0.0	0.0	0.0	0.0	0.0
**Fungivores**																
*Aglenchus*	Fu2	0.0	0.0	0.0	0.0	0.0	0.0	0.0	0.0	0.0	10.4	0.0	0.0	0.0	1.1	2.0
*Aphelenchoides*	Fu2	0.2	0.2	1.4	0.0	13.1	2.2	2.0	0.0	0.0	0.0	0.0	0.0	0.2	5.1	13.7
*Aphelenchus*	Fu2	4.2	14.8	15.6	10.1	0.2	10.1	17.4	7.7	15.6	4.2	10.8	8.3	4.5	7.1	3.1
*Axonchium*	Fu5	0.0	0.0	0.2	0.0	0.0	0.0	0.0	0.0	0.0	0.0	0.0	0.0	0.0	0.0	0.0
*Belondira*	Fu5	0.0	0.0	5.7	0.0	0.0	0.0	0.0	0.0	0.0	0.0	0.0	0.0	0.0	0.0	0.0
*Ditylenchus*	Fu2	0.0	0.0	0.0	0.0	0.0	0.2	0.7	0.0	0.0	0.0	0.0	0.0	0.0	0.0	0.0
*Doryllium*	Fu4	1.1	0.2	8.7	14.8	0.6	2.4	8.2	12.8	2.2	2.7	3.1	3.7	7.6	3.1	0.3
*Filenchus*	Fu2	1.3	0.0	4.9	2.4	14.5	0.9	6.4	4.3	1.7	1.5	4.3	2.9	3.7	4.7	3.6
*Oxydirus*	Fu5	0.0	0.0	0.5	0.5	4.1	0.0	0.0	0.0	0.0	0.4	0.6	1.0	1.5	0.2	1.5
*Tylencholaimellus*	Fu4	3.8	2.4	0.3	2.6	3.9	0.9	0.6	8.4	1.1	0.0	1.9	3.0	3.2	0.5	1.3
*Tylencholaimus*	Fu4	0.0	0.0	0.2	0.0	3.1	1.6	0.3	0.9	0.7	0.0	0.0	0.0	0.0	0.0	0.0
*Tylenchus*	Fu2	0.0	0.2	1.4	4.6	5.9	0.0	0.9	0.5	0.5	0.0	1.3	2.2	0.3	0.5	0.0
*Diphtherophora*	He2	0.0	0.0	0.8	0.0	0.2	0.0	0.4	0.0	0.0	0.0	0.0	0.0	0.0	0.0	0.0
**Herbivores**																
*Enchodelus*	He4	0.7	1.1	0.0	7.7	0.4	0.0	0.0	0.0	0.0	0.0	1.1	0.0	0.0	0.0	0.0
*Helicotylenchus*	He3	11.1	4.2	5.1	3.6	0.0	2.1	0.0	14.9	24.2	12.2	0.7	0.9	3.1	1.7	3.8
*Hemicriconemoides*	He3	0.0	0.0	0.0	0.5	0.0	0.0	0.0	0.0	0.2	0.0	0.0	0.0	0.0	0.0	0.0
*Hoplolaimus*	He3	0.4	0.0	0.1	0.0	0.0	0.0	0.0	0.0	0.7	0.0	0.0	0.0	0.0	0.0	0.0
*Longidorus*	He5	0.9	0.3	0.0	0.0	0.0	0.0	0.0	0.0	0.0	0.0	0.0	0.0	0.0	0.0	0.0
*Merlinius*	He3	0.0	0.0	0.0	0.2	0.0	0.0	0.0	0.0	0.3	1.2	1.2	0.0	0.0	0.0	0.0
*Oriverutus*	He4	0.0	0.0	0.5	0.0	0.0	0.0	0.0	0.0	0.0	0.0	0.0	0.0	0.0	0.0	0.0
*Paralongidorus*	He5	0.0	0.2	0.0	0.0	0.0	0.0	0.0	0.0	0.0	0.0	0.0	0.0	0.0	0.0	0.0
*Pratylenchus*	He3	20.8	0.0	1.6	1.5	0.0	0.0	0.2	15.8	4.4	3.9	0.5	1.4	0.0	0.9	0.0
*Psilenchus*	He2	1.1	0.7	1.3	0.7	2.0	0.0	0.9	0.0	0.4	0.0	0.7	0.3	0.2	0.2	0.4
*Rotylenchus*	He3	0.1	9.0	1.4	0.0	2.9	0.0	0.2	0.0	0.3	0.0	0.0	0.0	0.0	0.3	0.0
*Tylenchorhynchus*	He3	0.0	0.0	1.3	0.2	0.2	0.0	0.0	0.2	3.3	0.1	0.0	0.0	0.1	0.0	0.0
**Omnivores**																
*Crassolabium*	Om4	0.0	0.0	0.0	0.2	0.0	0.0	1.3	1.7	0.0	0.0	0.0	0.0	0.0	0.0	0.0
*Eudorylaimus*	Om4	2.1	4.8	1.8	1.7	7.6	0.0	3.4	0.0	0.9	2.6	4.3	0.8	1.0	2.8	3.8
*Mesodorylaimus*	Om4	0.6	0.3	2.4	0.2	2.5	7.9	1.7	1.9	0.0	0.1	1.5	0.0	0.0	0.5	0.0
*Microdorylaimus*	Om4	0.0	0.0	0.0	0.0	1.5	0.0	0.0	0.0	0.0	0.0	0.0	0.0	0.0	0.0	0.0
**Predators**																
*Aporcelaimellus*	Pr5	3.8	2.2	0.4	0.7	0.0	0.6	1.5	2.3	1.4	3.2	10.7	2.2	1.4	1.7	2.8
*Clarkus*	Pr4	1.4	0.0	1.4	0.7	0.2	0.2	0.0	0.9	0.0	0.4	0.4	1.3	0.8	0.3	1.5
*Discolaimoides*	Pr5	0.0	0.0	0.0	0.0	0.0	0.0	0.0	0.0	0.3	0.3	0.0	0.0	0.0	0.0	0.0
*Discolaimus*	Pr5	0.0	0.0	0.0	0.7	0.0	0.0	0.0	0.0	0.0	1.8	2.1	1.1	0.1	0.0	0.1
*Ironus*	Pr4	0.0	0.2	0.0	0.0	0.0	0.0	0.0	0.0	0.0	0.0	0.0	0.0	0.0	0.0	0.0
*Metaporcelaimus*	Pr5	0.0	0.0	0.2	0.0	0.0	0.0	0.2	0.0	0.0	0.0	0.0	0.0	0.0	0.0	0.0
*Moshajia*	Pr5	0.0	0.0	0.0	0.5	0.0	1.3	1.8	0.2	0.7	0.0	0.2	0.0	0.0	0.0	0.0
*Mylonchulus*	Pr4	1.1	5.2	2.7	3.8	0.3	0.0	0.4	0.0	1.1	6.2	2.8	1.9	3.1	1.0	3.8

^a^Elevation in metres above sea level.

^b^Functional guild is composite of trophic group and cp value: *Ba* Bacterivore, *Fu* Fungivore, *He* Herbivore, *Om* Omnivore, *Pr* Predator. Trophic groups and cp values are after Yeates et al. (1993) and Bongers (1988), respectively.

### Nematode indices and metabolic footprint along elevation gradient

**Figure 5:**
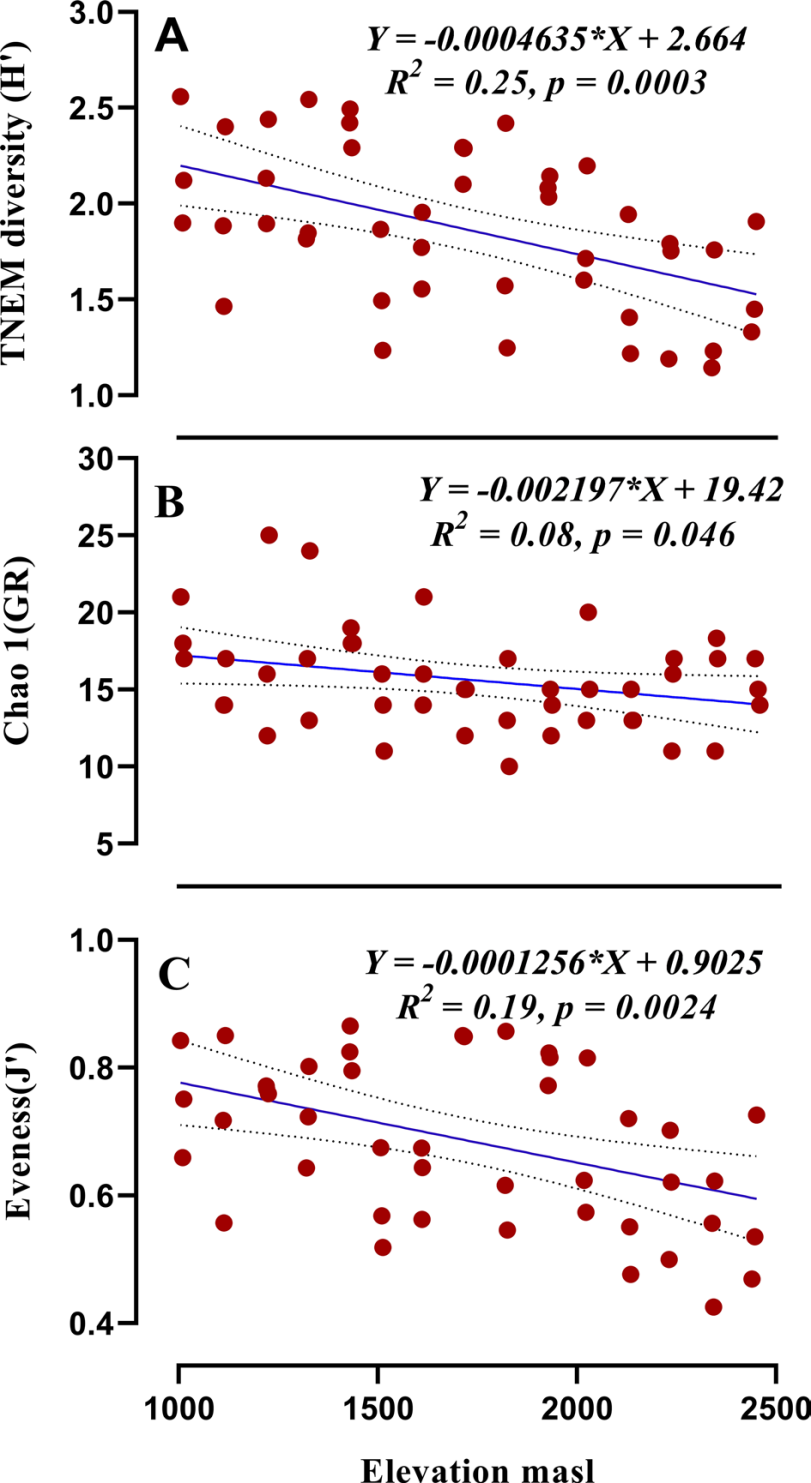
Relationship between elevation and total nematode diversity (**A**), generic richness (**B**) and evenness (**C**). Equation for *Y*, *R*^2^ (coefficient of determination), *p* (significance) of linear regression, regression line and dotted lines of 95% of confidence interval are displayed.

**Figure 6:**
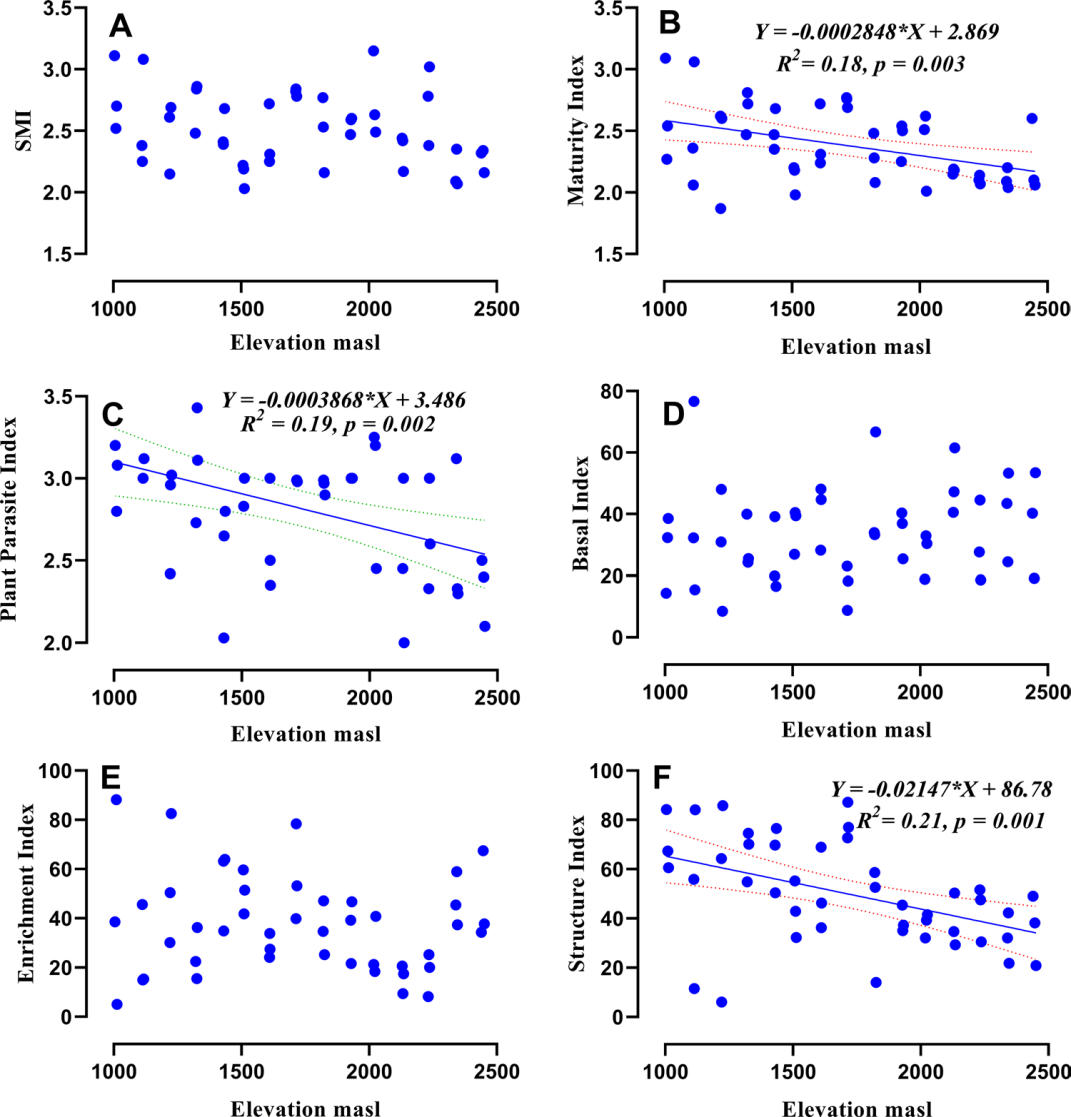
Relation between elevation and ΣMI (**A**), MI (**B**), PPI (**C**), BI (**D**), EI (**E**) and SI (**F**). Equation for *Y*, *R*^2^ (coefficient of determination), *p* (significance) of linear regression, regression line and dotted lines of 95% of confidence interval are displayed only for significant relationships.

**Figure 7:**
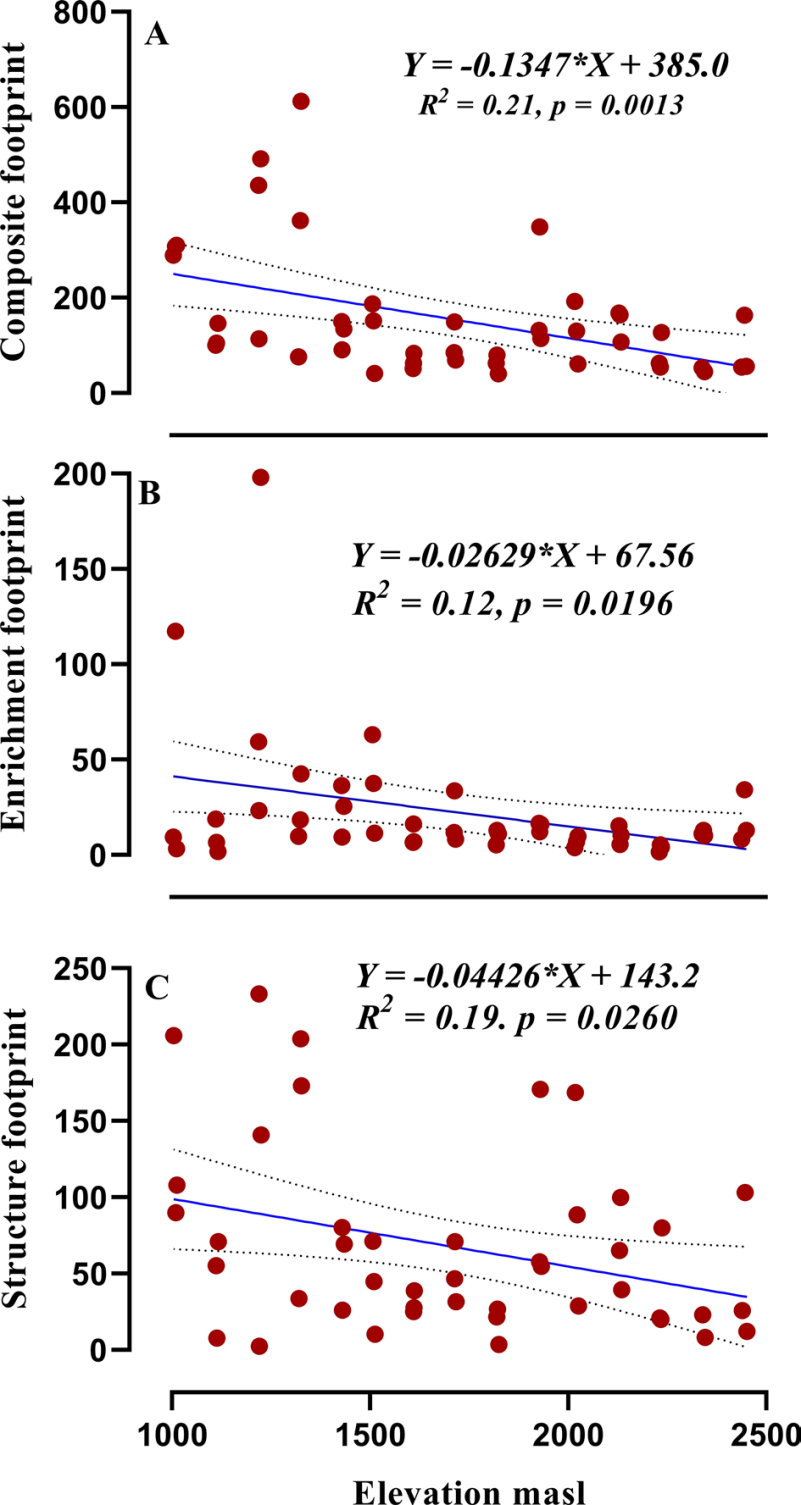
Relationship between elevation and composite metabolic footprint (**A**), enrichment footprint (**B**) and structure footprint (**C**). Equation for *Y*, *R*^2^ (correlation coefficient), *p* (significance) of linear regression and regression line are displayed.

All measured alpha diversity indices reflected a significant negative (Table 1) correlation with the increase of elevation. The values of H (*r* = − 0.49, *df* = 43), GR (*r* = − 0.32, *df* = 43) and J (*r* = − 0.42, *df* = 43) negatively correlated with increasing elevation (Fig. 5A–C). The nematode community MI (Fig. 6B), PPI (Fig. 6C) and SI (Fig. 6F) decreased from lower elevation to higher elevation. However ΣMI (Fig. 6A), BI (Fig. 6D) and EI (Fig. 6E) remained unaffected with change in elevation. The composite metabolic footprint, enrichment footprint and structure footprint all decreased with increasing elevation (Fig. 7A–C) indicating the amount of carbon entering the soil food-web from total nematode assemblages.

### Relationship of soil nematode assemblages with soil physiochemical properties

**Figure 8:**
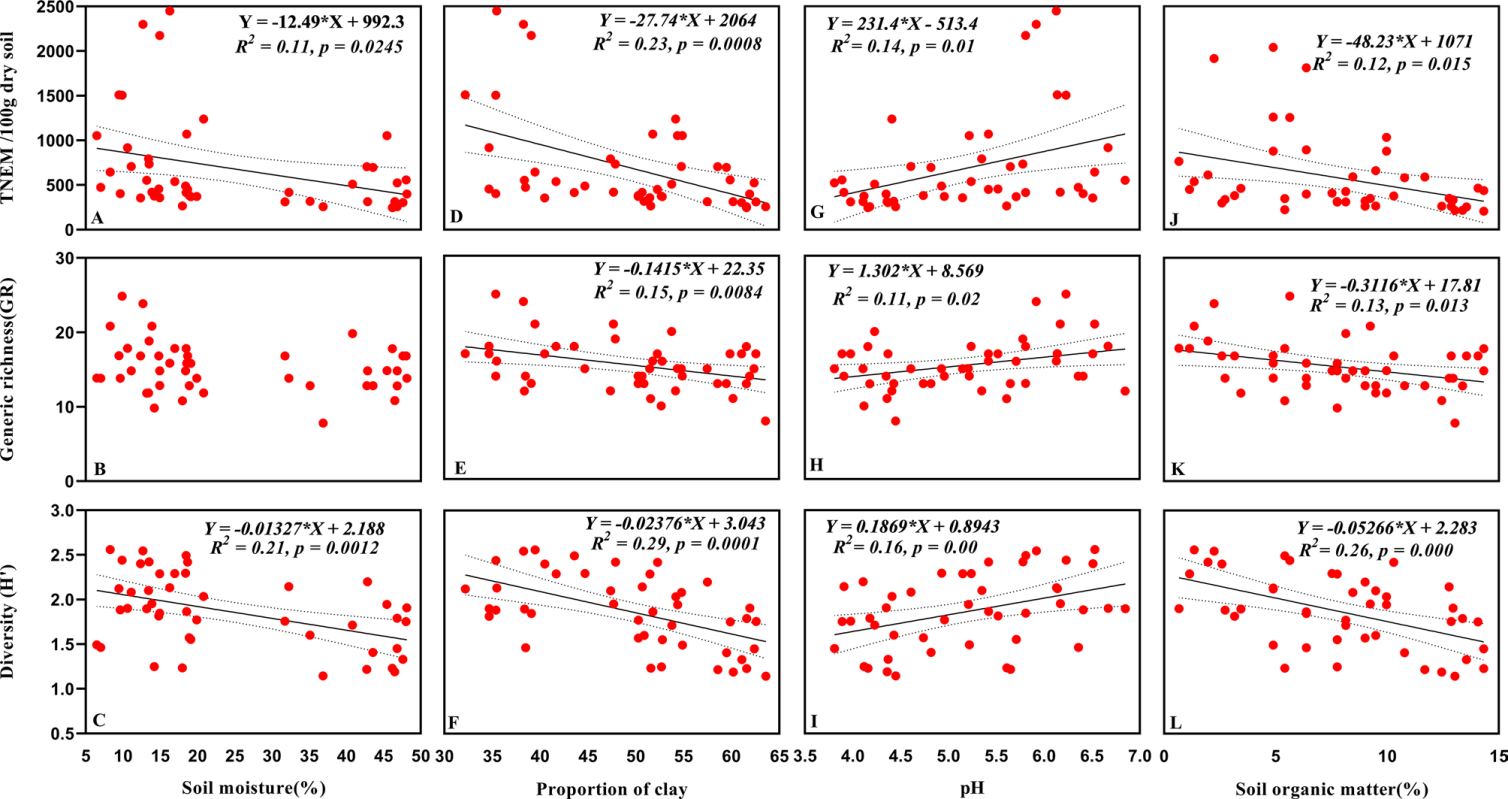
Relationship of soil moisture, proportion of clay, pH and soil organic matter with total nematode abundance, GR and H′ (**A**–**L**). Equation for *Y*, *R*^2^ (coefficient of determination), *p* (significance) of linear regression, regression line and dotted lines of 95% of confidence interval are displayed only for significant relationships.

**Figure 9:**
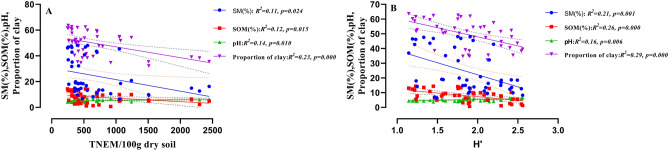
Relationships between four soil abiotic factors with nematode abundance (**A**) and diversity (**B**) estimated from a multiple linear regression model. Circle, square, triangle and inverted triangle represent SM%, SOM%, pH and proportion of clay respectively. R^2^, p value and doted lines representing 95% confidence interval of the best fit line are shown.

Total nematode abundance was negatively correlated with soil moisture (Fig. 8A) and proportion of clay (Fig. 8D) and positively correlated with pH (Fig. 8G). No significant correlation was observed between soil moisture and nematode generic richness (Fig. 8B). However, generic richness negatively correlated with proportion of clay (Fig. 8E) and positively correlated with pH (Fig. 8H). Soil moisture (Fig. 8C) and proportion of clay (Fig. 8F) showed negative influence on diversity (H′), whereas, pH showed positive influence on diversity (Fig. 8I). Soil organic matter showed a negative correlation with abundance, generic richness and diversity (Fig. 8J–L). Further, multiple regression analyses showed a high correlation between proportion of clay with abundance (*R*^2^ = 0.23,* p* = 0.000) and diversity (*R*^2^ = 0.29,* p* = 0.000) of nematodes as compared to other soil abiotic factors (Fig. 9A,B).

## Discussion

Elevation is a complicated, indirect gradient along which several environmental variables shift, resulting in a fundamental diversity gradient trend in animal and plant biogeography^.^ The temperate vegetation in higher altitudes of Banihal-Pass of Pir-Panjal mountain range experiences extremely harsh climatic conditions with almost half of the precipitation received in the form of snow, because soil remains deep frozen in winter months and experiences significant temperature fluctuations.

In line with our first hypothesis here we observed that total soil nematode abundance and diversity exhibit a significant decline with an increase in altitude which is consistent with the results of some earlier studies on nematodes^21,22^ and with other soil fauna including springtails and mites^25^, and other microarthropods^26,27^. In contrast to this, various patterns are found to occur in nematode community along the elevation gradient, at mid-elevation maximum diversity and species richness have been documented^19,28^, in alpine grasslands abundance and diversity increased with elevation^20^. However, similar studies on nematodes along gradient did not find any definite pattern^23,24^. As mentioned earlier, mountains in Pir-Panjal range tend to have greater climatic adversities that alter soil conditions, and these altered soil conditions explains the survival of sparse and coexistence of fewer nematode species in the higher elevations. Several environmental gradients are held responsible for shaping soil nematode communities. Among the environmental conditions that shift strongly with elevation is temperature, which gradually decreased with elevation (Supplementary Fig. S1) and proved detrimental for nematode community like other soil organisms. Nematodes are very sensitive to temperature changes with optimal of 20–25 °C for the survival and propagation, any change in temperature from optimum value leads to significant inhibition in nematode population^29–31^. In our study, temperature was below optimum possibly affecting soil nematode abundance and diversity as conditions were much harsher at higher elevations. Increased soil organic matter and moisture along the elevation gradient (Fig. 1) had a negative impact on nematode abundance and diversity, although they are considered as positive drivers of their community^29,32,33^. Several reasons can be held responsible for shaping this pattern of nematode abundance and biodiversity: (1) more proportion of clay in soil at higher altitude reduce the porosity of soil and restrict the movement of nematodes. This can also be explained by a previous study which implies that less abundant and small sized nematodes are favoured in clay soils^34^; (2) at higher elevation more precipitation is received, thus high soil moisture content may leads to formation of anaerobic conditions in the upper forest soils, potentially reducing the abundance and biodiversity. This negative correlation of soil moisture content is in line with a previous study^21^; (3) low temperature at high altitudes reduces microbial activity and decreases decomposition rate under coniferous vegetation canopy which is rich in lignin^35^ that may leads to accumulation of soil organic matter and made it unavailable to nematodes. The reason for the decrease in soil pH with elevation may be due to the litter accumulation that negatively effects the nematode community, as found in other soil biota^36^. Additionally our NMDS model revealed that different elevations can not harbour similar abundance of nematodes possibly due to environmental filtering, poor environmental conditions and resource heterogeneity.

Variation in nematode abundance belonging to different trophic groups were also observed. Abundance of herbivores, fungivores and omnivores significantly declined with altitude which is in line with Veen et al.^37^. A potential explanation for these findings is that herbivore nematodes are favoured in soils colonized by diverse assemblages of plants with well-developed fine root systems. At higher altitudes vegetation is sparse under forest canopy, possibly depriving food for herbivores which is in accordance with another studies that reported a reduction in the abundance of plant-feeders with altitude^12,38^. For herbivore nematodes, bottom-up effects from plant communities are thought to be more important than top-down pressure from predators^39^. Elevation has no effect on bacterial feeding nematodes in our study, which may be due to basal characteristics of bacterial feeders in food web that are mostly dominated by basal fauna (Ba_2−_ nematodes) which are stress tolerant and avoid stress by various adaptations. However, another studies^20,29^ reported a definite pattern of increase in bacterivore nematode from lower to higher elevations. The non-significant decline of predatory nematodes could be due to their key position in the soil food web, where they can switch on to different prey based on availability. Similar results were reported by Kergunteuil et al.^20^.

Maturity index measures the level of disturbance in the soil ecosystem, lower values indicating more disturbances. Sigma maturity index (ΣMI) takes in to account all nematode c-p groups and trophic groups found in a soil sample. Our result showed that ΣMI is not affected by elevation which contradicts the finding of earlier study^20^, which suggested that the increase in ΣMI was mainly driven by persister herbivore nematodes. However, in present study the decrease in MI, SI and PPI was found, which indicates lesser tolerance of K-selected free-living and pant parasitic nematodes towards stress conditions that are encountered at higher elevations. Furthermore, two nematode indices EI and BI, both of which takes in to consideration r-selected free-living nematodes which are tolerant to disturbances, remained unaffected by elevation which is in line with pervious finding^20^. In the present study, several nematode functional indices [maturity index (MI) structure index (SI) and plant parasitic index] generally decreased with increase in altitude. These results indicated that higher elevations harbour lesser complex and structured soil food webs, with fewer connections than those supported in lower elevations.

The metabolic footprint provides information on magnitude of carbon and energy flow in soil food webs^14^. The nematode metabolic footprint consists of composite metabolic footprint, enrichment footprint and structure footprint which are the representatives of whole nematode community^14^. Consistently with diversity, abundance, biomass and metabolic footprint of total nematodes decreased with elevation which is according to our second hypothesis. From this, it is implied that carbon assimilation in soil food web from autotrophic organisms decreased with elevation as found in composite metabolic footprint, enrichment footprint and structure footprint. The enrichment footprint is considered to be an indicator of resource entry into the soil food web^41^. The decline of enrichment footprint with elevation showed less entry of resources in the soil food web at high elevation because of slower decomposition rates influenced by low temperature and winter permafrost. Structure footprint reflects metabolic activity of higher trophic level nematodes, which have regulatory function in the food web^42,43^. The decrease in structure footprint with elevation may be related to decrease of resource entry in the food web at higher elevation, indicating a possible bottom-up control of the soil food web. Overall, this decrease in carbon assimilation of whole nematode community could be attributed to retention of more carbon in tree biomass than in soil of these temperate forests at higher elevation, although productivity is high^44^. However, decline in the efficiency of carbon to enter the soil faunal food web through nematodes with increasing altitude may be due to decrease in the availability of amplifiable prey for nematodes. Thus, for the mitigation of elevated atmospheric carbon, high altitude forest soils cannot be relied. Additional expansion of lower altitude forests in these regions need to be considered for mitigating and stabilising the elevated atmospheric carbon in soil food webs. But concerning global warming the upslope shifts in biodiversity are predicted due to temperature change. Keeping this in view, in future we expects larger nematode metabolic footprint at higher elevation, which indicates more carbon inflow in soil nematode community with the predicted change in temperature according to global warming. According to our second hypothesis, i.e. nematode metabolic footprint are the suitable indicators of elevation change, suggests that whether carbon is being partitioned in the soil food web or not, but its estimation alone will be valuable in predicting the environmental change caused my global warming.

Our results showed that nematode communities are not consistent with the elevation gradient under temperate vegetation in spring (April). Future studies could be done in different seasons of the year which could address the question that whether this elevation pattern in soil nematode community is general in this mountain range.

## Conclusion

Our findings from this study indicated that a more abundant and diverse nematode assemblage are supported at lower elevations and keep diminishing along elevation gradient under temperate vegetation cover in Banihal-Pass of Pir-Panjal mountain range. Temperature and local soil microhabitat are the main elevation induced environmental factors which shape the pattern of nematodes. We also observed that the role played by nematodes in carbon cycling decreases with elevation as the carbon footprint of nematodes showed significant decline. However, as elevation gradients are complex in mountain environments, further studies focused on vegetation type, temporal dynamics and biotic interactions of nematodes are needed to validate, whether this pattern is general.

## Methods

### Study site

The study site was situated in Banihal-Pass (33.4370° N, 75.1939° E) of Pir-Panjal mountain range. The region owing to its topography falls in typical temperate zone with maximum elevation of 2832 m. The area experiences a mean annual temperature and precipitation of 15.1 °C and 1363 mm respectively. However, change in temperature with elevation was calculated by lapse rate of 0.6 °C/100 m^19^ using an annual base temperature of 15.1 °C of the study site (Supplementary Fig. S1). At higher altitudes significant amount of precipitation is received in the form of snowfall. The climate varies with altitude (IMD 2014). The soils of the region are organic rich brown forest soils and podozolic soils. The vegetation cover at altitudes between 1000 and 2000 masl is of temperate deciduous type mostly dominated by *Pinus roxburghii, Robinia pseudoacacia, Populus* spp.,* Ulmus villosa, Aesculus indica, Juglans regia, Salix* spp. The zone between 2000 and 2500 masl is of temperate coniferous vegetation having predominance of *Albies pindrew, Pinus welichiana* and *Cedrus deodara.* Above 2500 masl, the vegetation cover is mostly alpine tundra and alpine pastures.

### Soil sampling

Sampling was conducted in the month of April 2018. A total of 45 soil samples were collected from 15 elevations along a mountain separated from each by ~ 100 m of elevation. At each elevation, three separate quadrats of 5 m^2^ were selected, spaced approximately 50 m apart in a horizontal line. From each quadrat five sub-samples, four from corners and one from centre at a depth 0–15 cm were combined to form a composite sample of 500 g. These samples were transported to lab within 2 days.

### Soil analysis

Physicochemical analysis of soil: about 350 g of the soil out of the initial bulk was used for measuring soil properties (soil moisture, soil organic matter, pH, and soil texture). Soil moisture was measured gravimetrically by drying at 40 °C for seven days. SOM was estimated following loss on ignition method^43^. Soil pH was measured by forming a suspension of 20 g of soil in 40 ml of deionised water using pH meter (ECPHTUTOR). Soil texture was calculated as the relative proportion of sand, silt, and clay expressed as percentage.

### Nematode extraction and identification

From 100 g of fresh soil sample, nematodes were extracted via decanting and sieving following the Cobb's method^45^. Each soil sample was put into a 1 l beaker and mixed with tap water. The water suspension was stirred and decanted into another 1 l beaker through 2 mm mesh sieve to remove stones and large debris. The water suspension was mixed further and then decanted through 53 μm mesh sieve. The material left on the sieve was collected in 250 ml beaker and further extraction was carried out by Baerman’s funnel method. Nematodes were removed for 2 days, stored at 4 °C, fixed in TAF and counted using inverted microscope (Olympus SZX10). Additionally, 200 individuals per sample were identified to generic level using compound microscope (Olympus BX41), with the aid of various texts^46–50^. The genera identified were also assigned to different functional guilds^41,51^.

### Community analysis

Nematode abundance and trophic abundance was adjusted as total number of individuals in 100 g of dry soil. Alpha diversity of nematodes were calculated by Shannon index ($H = - \sum\nolimits_{i = 1}^{n} {P_{i} } \ln P_{i}$), generic richness, Chao1 = *S* + *F*_1_(*F*_1_ − 1)/(2(*F*_2_ + 1)) (where S is number of genera; F1 and F2 indicate genera represented by one and two individuals of a genus in a sample respectively) and evenness index (*J* = *H*/ln*S*); indices were analysed with PAST 3.26^52^. Six nematode community based ecological indices were calculated: sigma maturity index (ΣMI)^52^, maturity index (MI)^52^, plant parasitic index (PPI)^53^, basal index (BI)^41^, enrichment index (EI)^41^ and structure index (SI)^41^.

The metabolic footprints which estimates magnitude of ecosystem services and functions provided by nematodes to the soil food web were calculated using the equations:

$$W = \left( {D^{{2}} \times L} \right)/\left( 1.6 \times 10^{{6}} \right)^{54},$$

$$F = \Sigma (N_{t} (0.1\left( {W_{t} /m_{t} } \right) + 0.273(W_{t} 0.75))^{14},$$

where *W* is the nematode biomass (μg), *D* and *L* are maximum body diameter (μm) and body length (μm) respectively, *N*_*t*_ is the number of nematodes in genus *t*, *W*_*t*_ is the estimated body weight of genus *t*, and *m*_*t*_ is the c-p group of the genus *t*. Metabolic footprints and nematode ecological indices were calculated using the NINJA online program^55^.

### Statistical analysis

Spearman correlation and simple linear regression was used to study the effect of elevation on abiotic factors, soil nematode community as well as relationship of soil abiotic factors with soil nematode communities. All statistical analysis and graphs were prepared with the help of software GraphPad Prism8.0.2^56^. *P* was considered significant below 0.05. Analysis of four abiotic factors of 45 soil samples was correlated with elevation and tested with simple linear regression. We performed multiple linear regression models to analyse the relationships between soil abiotic factors and nematode abundance and diversity. Non-metric multidimensional scaling (NMDS) ordination plot based on Bray–Curtis similarity measures was produced to visualize the patterns of nematode communities. The one-way analysis of similarity (ANOSIM) was used to compare nematode community structure between elevations, based on the square root transformed abundance of nematode genera using the software PAST 3.26^51^.

## Supplementary Information

Supplementary Figure 1.

## Abbreviations

masl: Meters above sea level
MAT: Mean annual temperature
IMD: Indian Meteorological Department
SOM: Soil organic matter
TAF: Triethanolamine formaldehyde
TNEM: Total nematode
