# Supplementary material for: Altitudinal gradient affect abundance, diversity and metabolic footprint of soil nematodes in Banihal-Pass of Pir-Panjal mountain range
Source: Sci Rep. 2021 Aug 10;11:16214. doi: 10.1038/s41598-021-95651-x (PMC8355321; doi:10.1038/s41598-021-95651-x)
Supplement: Supplementary file 1 — Supplementary Figure 1. [file 41598_2021_95651_MOESM1_ESM.pdf]

Altitudinal gradient affect abundance, diversity and metabolic footprint of soil nematode in Banihal-Pass of Pir-Panjal mountain range.

Shahid Afzal, Humira Nesar, Zarrin Imran and Wasim Ahmad

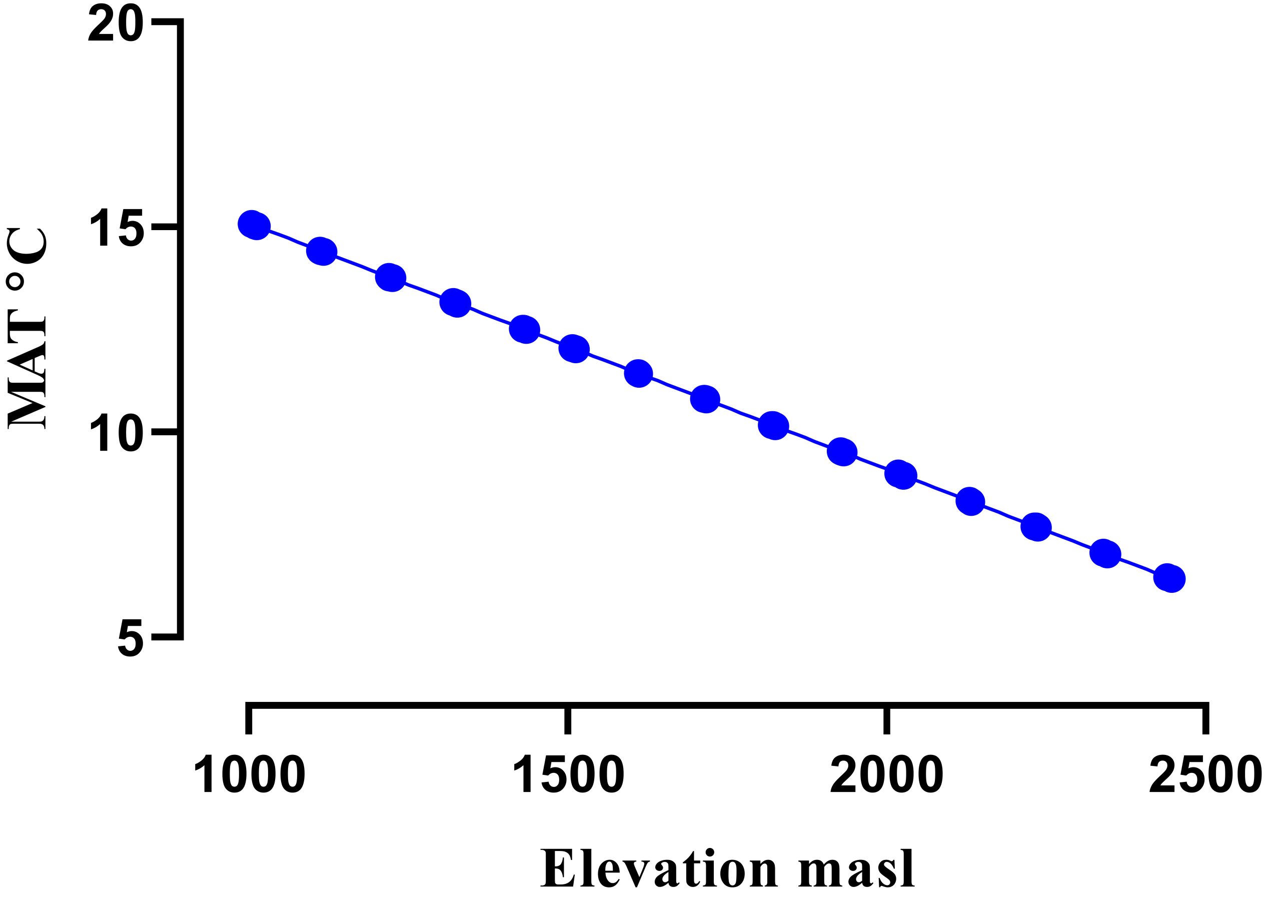

Fig. S1: Effect of elevation on Mean Annual Temperature (℃).
